# Ultrasound Nondestructive Evaluation (NDE) Imaging with Transducer Arrays and Adaptive Processing

**DOI:** 10.3390/s120100042

**Published:** 2011-12-22

**Authors:** Minghui Li, Gordon Hayward

**Affiliations:** Centre for Ultrasonic Engineering, Department of Electronic and Electrical Engineering, University of Strathclyde, 204 George Street, Glasgow G1 1XW, UK; E-Mail: g.hayward@eee.strath.ac.uk

**Keywords:** transducer array, adaptive beamforming, ultrasound imaging, non-destructive evaluation (NDE)

## Abstract

This paper addresses the challenging problem of ultrasonic non-destructive evaluation (NDE) imaging with adaptive transducer arrays. In NDE applications, most materials like concrete, stainless steel and carbon-reinforced composites used extensively in industries and civil engineering exhibit heterogeneous internal structure. When inspected using ultrasound, the signals from defects are significantly corrupted by the echoes form randomly distributed scatterers, even defects that are much larger than these random reflectors are difficult to detect with the conventional delay-and-sum operation. We propose to apply adaptive beamforming to the received data samples to reduce the interference and clutter noise. Beamforming is to manipulate the array beam pattern by appropriately weighting the per-element delayed data samples prior to summing them. The adaptive weights are computed from the statistical analysis of the data samples. This delay-weight-and-sum process can be explained as applying a lateral spatial filter to the signals across the probe aperture. Simulations show that the clutter noise is reduced by more than 30 dB and the lateral resolution is enhanced simultaneously when adaptive beamforming is applied. In experiments inspecting a steel block with side-drilled holes, good quantitative agreement with simulation results is demonstrated.

## 1. Introduction

**Figure 1.**
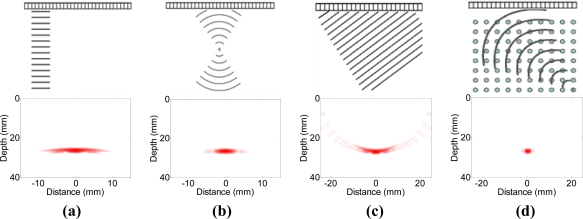
Schematic diagrams of conventional inspection methods, and images obtained for a single point reflector. **(a)** plane scan, **(b)** focused scan, **(c)** sector scan, **(d)** total focusing method. Point reflector is located on centre line of array and at a distance of 25 mm. Dynamic range is 40 dB.

Quantitative characterizations of materials and structures by non-invasive means are essential in a wide range of applications like flaw detection, structure health monitoring, materials characterization, and *etc.* [1]. The applications of transducer array systems to ultrasound non-destructive evaluation (NDE) have increased dramatically in recent years, due to the great advantages of enhanced coverage, sensitivity and flexibility, where multiple inspections can be performed without the need for reconfiguration [2]. Through staggered firing of transmitter elements, plane beams, focused beams and steered beams can be formed to generate real time images, as illustrated in Figure 1. On the other hand, if the complete set of time domain data from all combinations of transmitter and receiver elements is acquired with full matrix capture (FMC), the total focusing method (TFM) [3] can be used as an offline imaging technique. Through dynamic focusing in both transmission and reception at every point in the target region, higher quality images can be created with improved resolution and contrast for enhanced sensitivity and greater coverage [4,5].

In all the aforementioned ultrasonic imaging techniques, beam steering and focusing are achieved with delay-and-sum operations, where the signals received by the array elements are delayed to compensate the expected time-of-flight differences to each array element (focal laws), and then added together. However, the image resolution, contrast and dynamic range achievable with this method are fundamentally limited. Along the years, many research efforts have been devoted to find techniques that enhance the image quality, but some characteristics are often improved at the expenses of losses in some others. A typical example is pre-determined apodization with such as a Hanning or Hamming shaping function, which reduces the sidelobe level with an adverse effect on the lateral resolution [6]. In medical imaging with contrast being essential, this method is quite useful; however, in NDE applications with resolution being more relevant, apodization provides marginal or no benefits at all.

In recent works, advanced beamforming has been investigated for medical ultrasound imaging, where the received per-element delayed data samples are weighted with a data-dependent or spatially variant apodization vector prior to summation. This delay-weight-and-sum operation can be considered as applying an adaptive spatial filter to the received signals across the probe aperture to remove the interference and noise. The Capon adaptive beamformer was applied to compute the data-dependent weight vector by several authors, and demonstrated improved imaging contrast and resolution for simulated and phantom data [7–10]. Camacho *et al.* designed the weight vector by considering the aperture data phases explicitly in the image formation process [11]. Guenther and Walker calculated the weight function using the constrained least squares theory, and the weights were created with the goal of limiting the energy of the point spread function outside a certain area while maintaining a peak at the focal point [12,13]. Stankwitz *et al.* developed a spatially variant nonlinear apodization technique to find the optimal weight function, which used the lateral phase differences between the Hanning and uniformly weighted data to distinguish target signals from interference [14].

In NDE practice, most materials like concrete, stainless steel and carbon-reinforced composites used extensively in industries and civil engineering exhibit heterogeneous internal structure. When inspected using ultrasound, the signals from defects are significantly corrupted by the echoes form randomly distributed scatterers, even defects that are much larger than these random reflectors are difficult to detect with the standard delay-and-sum technique [15]. In this paper, we propose to use adaptive beamforming and spatial filtering in reception to enhance defect detection and NDE imaging. The adaptive weights are computed from the statistical analysis of the data samples based on the minimum variance theory, and the ways of increasing robustness in NDE scenarios are considered. In simulations with point reflectors, the technique reduces the clutter noise level by more than 30 dB compared with the delay-and-sum beamformer while simultaneously improving the lateral resolution. In experiments inspecting a steel block with side-drilled holes, good quantitative agreement with the simulation results is demonstrated. In order to compute the data-dependant weights, the method is more computation-intensive than delay-and-sum operations, but in many areas of industrial NDE, the target is static and it is reasonable to perform data analysis offline.

The outline of the paper is the following: Section 2 presents the method and describes how robustness is achieved through diagonal loading. The adaptive beamformer is evaluated against the gold-standard delay-and-sum beamformer on simulated and experimental datasets in Sections 3 and 4, respectively. Concluding remarks are drawn in Section 5.

## 2. Data Model and Problem Formulation

### 2.1. Data Model

Consider an arbitrary array of *N* transducer elements. Assume that there are *J* + 1 scatters in the target region, each reflecting a signal, *s_j_*(*t*), *j* = 0, …, *J*. The time series received at the *n*th element is:

(1) $$x_{n}\;(t)=\sum_{j=0}^{J}\;g_{n,j}\;s_{j}\;(t-\tau_{n,j})+v_{n}\;(t),\;\;n=1,\;\cdots ,\;N$$

where *g_n,j_* represents the amplitude adjustment corresponding to the distance from reflector *j* to sensor *n* and the gain of that sensor, *τ_n,j_* is the time delay from reflector *j* to sensor *n*, and *v_n_*(*t*) is the noise on channel *n*.

We assume that the array is focused on scatter 0 in reception through delaying each channel by *τ_n,0_*, *n* = 1, …, *N*. Under this assumption, *s*_0_(*t*) is the desired signal and the other reflectors are sources of interference. The time-delayed signal received at channel *n* can be described as:

(2) $$x_{n}\;(t)=g_{n,0}\;s_{0}\;(t)+\sum_{j=1}^{J}\;g_{n,j}\;s_{j}\;(t+\tau_{n,0}-\tau_{n,j})+v_{n}\;(t),\;\;n=1,\;\cdots ,\;N$$

The time-delayed array output vector, **x**(*t*), is given by:

(3) $$x\;(t)={\left[\begin{array}{cccc} x_{1}\;(t) & x_{2}\;(t) & \cdots & x_{N}\;(t) \end{array}\right]}^{T}$$

where [•]*^T^* denotes the transpose operator.

### 2.2. Problem Formulation and Adaptive Beamforming

**Figure 2.**
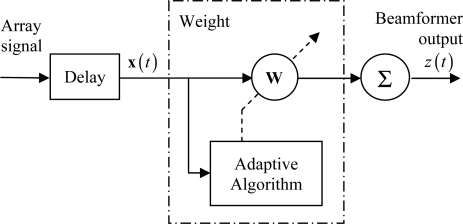
Schematic of adaptive beamforming.

As shown in Figure 2, adaptive beamforming is achieved by weighting the observations with data-dependent apodization, after each channel is appropriately delayed to focus at a point in the image. The output of the beamformer can be described as:

(4) $$z\;(t)={w\;(t)}^{H}\;x\;(t)$$

where:

(5) $$w\;(t)={\left[\begin{array}{cccc} w_{1}\;(t) & w_{2}\;(t) & \cdots & w_{n}\;(t) \end{array}\right]}^{T}$$

is a weight vector, and [•]*^H^* stands for the operator of conjugate transpose. The minimum variance beamformer seeks to minimize the power of the beamformed output *z*(*t*) while maintaining unit gain on the focal point [16]. This constrained optimization problem can be formulated as:

(6) $$\underset{w(t)}{\text{min}}\;{w\;(t)}^{H}\;R\;(t)\;w\;(t),\;\text{subject to}\;\;{w\;(t)}^{H}\;a=1$$

where:

(7) $$R\;(t)=E\;\left[x\;(t)\;{x\;(t)}^{H}\right]$$

is the data spatial covariance matrix, and:

(8) $$a={\left[1\;\;\;1\;\;\;\ldots \;\;\;1\right]}_{N}^{T}$$

is a *N* × 1 column vector of ones, because the data have already been delayed to focus on the point of interest. The optimal weight vector is the solution to (6), given by:

(9) $$w_{\mathrm{opt}}\;(t)=\frac{{R\;(t)}^{-1}\;a}{a^{H}\;{R\;(t)}^{-1}\;a}$$

In practice, the ideal covariance matrix **R**(*t*) (7), which is computed from infinite number of data samples, used in (9) is not available, and has to be replaced by the sample covariance matrix. If the full matrix capture data acquisition is used, the estimate can be obtained by:

(10) $$\hat{R}\;(t)=\frac{1}{N}\;\sum_{k=1}^{N}\;x^{k}\;(t)x^{k}\;{\left(t\right)}^{H}$$

where **x**^*k*^(*t*) is the delayed array output vector when the *k*th array element is excited in transmission and the array is focused onthe point of interest in reception.

The minimum variance beamformer is an optimal spatial filter that maximizes the array output signal-to-interference-plus-noise ratio (SINR) [17,18]. However, its performance degrades in the presence of mismatches or errors like wrong assumptions of acoustic velocity or phase aberrations. This phenomenon is referred to as signal self-nulling, since the mismatches lead to targets appearing slightly out of focus and the beamformer will treat them as interference and try to minimizethem [19–21]. There exists several methods for increasing the robustness at the expense of resolution. Diagonal loading [22] is a straightforwardbut effective approachin this application, where a constant, η, is added to the diagonal of the estimated covariance matrix:

(11) $${\hat{R}}_{\mathrm{DL}}\;(t)=\hat{R}\;(t)+\eta I$$

where **I** is a *N* × *N* identity matrix, and:

(12) $$\eta =\frac{1}{N}\;\mathrm{tr}\;\left[\hat{R}\;(t)\right]$$

where *tr*[•] denotes the trace operator. The amount of diagonal loading in (12) is proportional to the power in the received signals. However, *η* can be tweaked to make a trade-off between robustness and resolution, and a greater *η* leads to a more robust beamformer in (9), but the resolution is decreased.

## 3. Simulations

**Figure 3.**
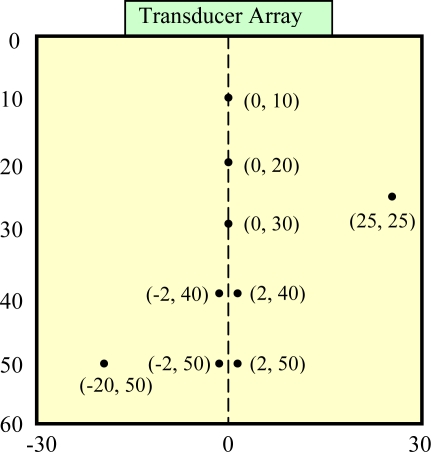
Schematic of simulated specimen.

In this section, we analyze the performance of the adaptive beamformer for interference and noise reduction with two simulations. Its capability to image point reflectors and resolve closely spaced emitters is assessed and compared against that of the delay-and-sum beamformer. Figure 3 shows the schematic of the simulated specimen, in which multiple single and paired point reflectors are located at different depths and distributed laterally. The material is assumed to be ideal, homogeneous and lossless with the wave propagation speed *c* = 6,300 m·s^−1^. A 5 MHz linear transducer array is utilized in contact with the specimen upper surface, which consists of 64 omni-directional elements with the inter-element spacing 0.63 mm that is exactly the half wavelength in the medium at the centre frequency of the transducer. The output signal of each element is a five cycle, Gaussian windowed tone burst with a centre frequency of 5 MHz and a −6 dB bandwidth of 50%. The reflectors have a normalized unity amplitude with white Gaussian noise being added to the signals, corresponding to a SNR = 20 dB for a single echo and channel. A sampling frequency of 100 MHz is used. The simulation parameters are summarized in Table 1.

**Figure 4.**
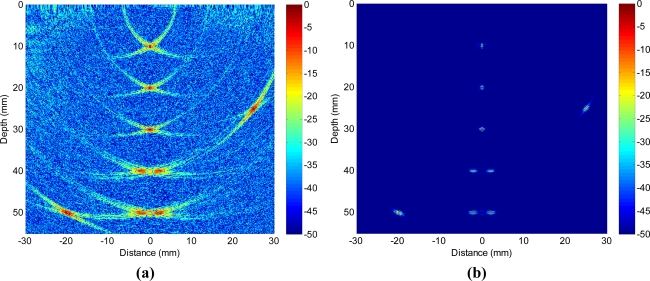
Simulated images with a 50 dB dynamic range obtained with **(a)** delay-and-sum beamforming, and **(b)** adaptive beamforming. A single element at array centre transmits and all array elements receive with dynamic focusing at every point in image.

To evaluate the performance of the adaptive beamformer as a spatial filter in reception, in the first example, we assume that a single element at the array centre transmits and the signals received by all array elements are dynamically focused at every point in the image in reception (one-way focusing). Figure 4 shows the achieved images with a 50 dB dynamic range. Figure 4(a) is obtained with the standard delay-and-sum approach, where the side lobe indications are quite visible together with significant clutter noise. The point reflectors appear to have certain shapes/size and the paired reflectors are unable to be separated. Due to the presence of strong clutter noise, it is difficult to detect smaller and weaker reflectors. On the contrary, Figure 4(b) shows the image obtained with adaptive beamforming with the same dynamic range. The noise has been completely removed (up to the level of −50 dB) and the side lobe indications have been significantly reduced, and all point reflectors are clearly seen with 2 paired reflectors being resolved.

**Figure 5.**
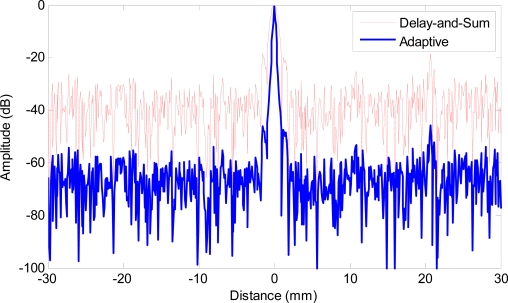
Lateral profiles of simulated images at range of 30 mm. Red solid line: delay-and-sum beamforming, blue bold line: adaptive beamforming.

To quantify the reduction on side lobe level and main lobe width, Figure 5 shows the lateral profiles of the images at the range of 30 mm that corresponds to one of the point reflectors. The red solid line shows the lateral profile obtained with delay-and-sum beamforming (−6 dB main lobe width being 1.12 mm), and the blue bold line shows the profile achieved with adaptive beamforming (−6 dB main lobe width being 0.22 mm). The reduction on main lobe width and side lobe level provided by adaptive processing is evident and significant, the side lobe level is reduced by more than 30 dB, and the −6 dB main lobe width is more than 80% smaller compared with that of delay-and-sum.

**Figure 6.**
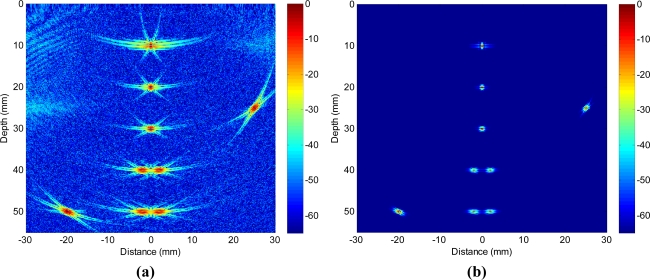
Simulated images with a 65 dB dynamic range obtained with **(a)** total focusing method, and **(b)** adaptive beamforming. Array is dynamically focused at every point in image on both transmission and reception.

**Figure 7.**
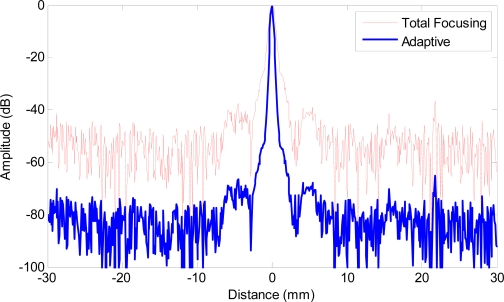
Lateral profiles of simulated images at range of 30 mm. Red solid line: total focusing method, blue bold line: adaptive beamforming.

In the second example, adaptive beamforming is evaluated against the total focusing method (TFM), which is able to achieve the best image quality among all standard inspection techniques as shown in Figure 1 through post processing of the received data samples. Figure 6 shows the images with a 65 dB dynamic range obtained by using TFM [Figure 6(a)] and adaptive beamforming in reception [Figure 6(b)] respectively, and the lateral profiles of the images at the range of 30 mm are depicted in Figure 7. When comparing Figure 6(a) with Figure 4(a) and Figure 7 with Figure 5 side-by-side, we may notice that the image quality is greatly improved, and the quantitative analysis of lateral profiles reveals that the clutter noise is reduced by about 15 dB and the −6 dB main lobe width is reduced from 1.12 mm to 0.86 mm, due to the capability of TFM to focus the array dynamically at every point in both transmission and reception.

However, the sidelobe indications are still pretty visible in Figure 6(a), especially in the regions close to the main lobe, which potentially hinder the detection of surrounding smaller or weaker reflectors and limit the capability to resolve clustered reflectors. When adaptive beamforming is applied, the image quality is significantly enhanced in terms of the clutter noise level, image resolution, contrast, and dynamic range as shown in Figure 6(b). The lateral profiles in Figure 7 indicate that the main lobe width is more than 50% smaller (−6 dB main lobe width of TFM being 0.86 mm and that of adaptive beamforming being 0.37 mm) and the clutter noise is reduced by more than 30 dB, due to the application of adaptive beamforming. The performance improvement observed in Example 2 is similar to that in Example 1, because the adaptive processing is applied in reception only in both examples.

## 4. Experimental Verification

**Figure 8.**
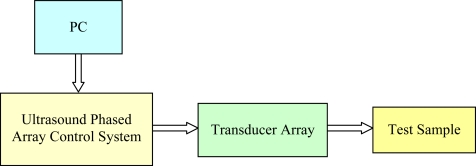
Schematic diagram of experimental apparatus.

**Figure 9.**
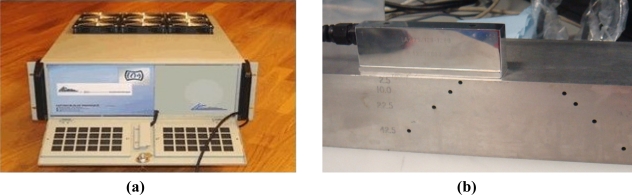
Experimental apparatus. **(a)** OPEN ultrasound phased array control system (LeCouer, France), **(b)** 5 MHz, 128-element linear transducer array (Vermon, France) and test sample of solid steel block with multiple side-drilled holes. Transducer array is in contact with sample upper surface with gel coupling.

The experimental apparatus employed in this work consists of three components, namely, the excitation and acquisition equipment, the ultrasound transducer array, and the test samples, as illustrated in Figure 8. The former is based on the OPEN ultrasound phased array control system (LeCouer, Chuelles, France), as shown in Figure 9(a), with 128 independent parallel channels. A personal computer connected to the OPEN system is used to control the excitation sequence and to store the received signals for further processing. A Matlab (The MathWorks, Natick, MA) routine is developed to implement full matrix capture data acquisition, *i.e.*, each array element is sequentially excited and the signals received by all the array elements are stored. In this way, a complete data set composed of *N*^2^ (*N* = 128) signals is obtained.

The test sample is made of a solid steel block with thickness of 60 mm in which multiple 3-mm diameter cylindrical side holes are drilled at different depths and lateral positions, as shown in Figure 9(b). A 5 MHz, 128-element linear transducer array (Vermon, Tours, France) with a 0.7 mm pitch is used in contact with the sample upper surface with gel coupling. A sampling rate of 40 MHz and a digital resolution of 16 bits are used. The parameters for the experiment are listed in Table 2.

**Figure 10.**
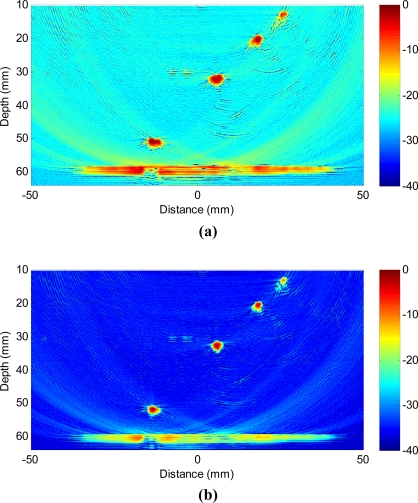
Experimental images with a 40 dB dynamic range obtained with **(a)** total focusing method, and **(b)** adaptive beamforming. Transducer array is dynamically focused at every point in image on both transmission and reception.

**Figure 11.**
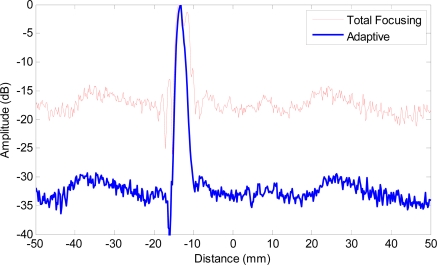
Lateral profiles of experimental images at range of 52 mm. Red solid line: total focusing method, blue bold line: adaptive beamforming.

Figure 10(a) shows the B-scan image obtained with TFM that allows the array to be dynamically focused at every point in the target region based on delay-and-sum operations on both transmission and reception. Figure 10(b) shows the image obtained with adaptive beamforming in reception. The first remarkable point is that not only the clutter noise is greatly reduced, but also the side-drilled holes are better characterized in shape and size. To quantify the performance enhancement, Figure 11 shows the lateral profiles of the experimental images at the range of 52 mm that corresponds to the lowest side-drilled holes. The red solid line shows the lateral profile obtained with TFM (−6 dB main lobe width being 3.74 mm), and the blue bold line shows the profile achieved with adaptive beamforming (−6 dB main lobe width being 1.83 mm). We may notice that the clutter noise level is reduced by about 15–20 dB, which leads to the enhanced contrast in the image.

Although the clutter noise reduction obtained in experiments (Figures 10 and 11) appears to be less than the 30 dB decrease in simulations as demonstrated in Figures 6 and 7, the improvement is of significantly practical interests and importance to identify weaker reflectors, especially in highly scattering materials. Better performance is observed in simulations because there are mismatches in the experiments between the ideal data model depicted in Equation (3) and the received signals due to the various distortions and errors like the heterogeneous propagation media, inaccurate wave speed, coupling variation, and *etc.* The performance will be enhanced if the adaptive beamformer is designed to be more robust to the model distortions. In addition, the images will be further improved if advanced beamforming is also applied in transmission.

## 5. Conclusions

In this paper, we present a novel approach for ultrasound NDE imaging, which applies minimum variance beamforming to the received signals and demonstrates significant performance improvement compared to delay-and-sum beamforming on both simulated and experimental data. Diagonal loading has been designed to increase robustness of the method. With adaptive filters applied spatially to the observations across the probe aperture, the spurious echoes and clutter noise are significantly reduced. This approach offers a great potential to inspect scattering materials, enhance sensitivity to smaller flaws, increase imaging resolution and contrast, and improve the definition of defect size and shape. Computation of the data-dependent weight vector for each pixel prohibits the method from real-time imaging, however, in many areas of industrial NDE the samples and defects are static and it is reasonable to carry out data analysis offline for more reliable, accurate and detailed defect characterization.

## Figures and Tables

**Table 1.** Simulation parameters.

**Simulation parameters**	**Value**
Number of elements	64
Element pitch	0.63 mm
Centre frequency	5 MHz
Bandwidth	50%
Element SNR	20 dB
Material	Lossless and homogeneous
Speed	6,300 m/s
Sampling frequency	100 MHz

**Table 2.** Experiment parameters.

**Experiment parameters**	**Value**
Number of elements	128
Element pitch	0.70 mm
Centre frequency	5 MHz
Material	Steel
Digital resolution	16 bits
Sampling frequency	40 MHz
